# Supplementary material for: Neonatal brain injury causes cerebellar learning deficits and Purkinje cell dysfunction
Source: Nat Commun. 2018 Aug 13;9:3235. doi: 10.1038/s41467-018-05656-w (PMC6089917; doi:10.1038/s41467-018-05656-w)
Supplement: Supplementary file 1 — Supplementary Information [file 41467_2018_5656_MOESM1_ESM.pdf]

Supplementary Information for:

"Neonatal brain injury causes cerebellar learning deficits and Purkinje cell dysfunction"

Aaron Sathyanesan[1,†], Srikanya Kundu[1,†], Joseph Abbah[1], and Vittorio Gallo[1,2]*

[1]Center for Neuroscience Research, Children's Research Institute, Children's National Medical Center, Washington DC, USA

[2]The George Washington University School of Medicine and Health Sciences, Washington, DC, USA

[†]Co-first authorship

*Corresponding author:

Vittorio Gallo

vgallo@childrensnational.org

SUPPLEMENTARY METHODS

Hypoxic rearing

Hypoxic rearing was performed as described previously[1, 2, 3, 4] with a CD-1 foster dam, between P3-P11. This time window in mice closely maps on to the time window during which the cerebella of premature human infants is especially vulnerable to hypoxic injury[5].

Erasmus Ladder experimental parameters and metrics analyzed

Broadly, ladder experiment were conducted in a similar manner as described in previous studies[6, 7, 8]. Each training trial consisted of a light cue followed by an air cue after which the mouse left the goal-box and traversed the ladder to the opposite goal-box. The "resting state interval" setpoint or the time the mouse spent in the goal-box between trials was set to 15 seconds with an interval randomization of ± 5 seconds, meaning that prior to any given trial, the resting state interval would randomly lie between 10 and 20 seconds. Once the animal left the goal-box and was on the ladder, a light automatic tail-wind was applied to motivate the animal to reach the opposite goal-box.

Previously used ladder metrics were analyzed[7, 9] to test for motor coordination and adaptive cerebellar learning. The Erasmus Ladder software was used to measure the following metrics:

1. Missteps: the percentage of steps whereby a mouse makes a faulty movement onto the lower misstep rungs (Figure 1b, dark blue rungs; 1c, grey rungs) rather than the default walking rungs (Figure 1b,c; light blue rungs).

2. Backsteps: the percentage of steps whereby a mouse makes a backward movement i.e. in the direction opposite to that of the tail-wind.

3. Short steps and long steps: the percentage of steps whereby a mouse makes a movement from one rung to the next rung on the same side (Figure 1b, "Short step") or the second next rung (Figure 1b, "Long step").

4. Pre-perturbation steptime: the time-difference (Δt) between subsequent rung activations immediately preceding the obstacle (Figure 1b, "Δt = Pre-perturbation") on the same side.

5. Post-perturbation steptime: the time-difference (Δt) between rung activation immediately preceding the obstacle (Figure 1b, "Δt = Post-perturbation") and that immediately following the obstacle on the same side. Both pre- and post-perturbation steptimes are measured on the order of milliseconds. During sessions 5 through 8, obstacles are automatically raised, randomly along a mouse's path during a given trial (one obstacle presentation per trial). Obstacle presentation consists of two steps – computer prediction of obstacle position based on mouse movement, and automated physical presentation of the obstacle. "Post-perturbation steptime" for sessions 1 through 4 (where there is no physical perturbation) were measured based on the first part of obstacle presentation which involves computer prediction of which obstacle to activate but without physically activating and presenting it. This metric serves as an internal control to compare within post-perturbation steptime measurements across all 8 sessions.

Adaptive cerebellar learning is tested by comparing ladder rung steptimes between Hx and Nx groups during training and challenge phases of the learning paradigm. While training sessions measure basal steptimes, challenge sessions measure steptimes when an obstacle (US) is presented, paired with a 250-millisecond preceding warning tone (CS). A higher post-perturbation steptime during session 5 indicates the start of associative learning, where mice are beginning to adapt their movements to avoid the obstacle. In subsequent sessions, normal mice learn to adapt their movements by associating the US and CS, increasing movement speed to avoid the obstacle, thus resulting in a decrease in post-perturbation steptimes.

Optogenetic targeting of PCs in the cerebellar cortex

Viral injections were performed stereotactically into *Pcp2-cre* (Mpin) animals at P9, at three injection sites in the simple lobule, bilaterally: anterior/posterior – 5.6 mm / -5.4 mm / -5.5 mm; medial/lateral -2.1 mm/ -2.0 mm/ 2.3 mm from the bregma; dorsal/ventral -0.35 mm from the dura. During the entire injection procedure, mice were placed under 2% isoflurane anesthesia in oxygen delivered from a precision vaporizer (Harvard Apparatus). A total of 1.0 μl viral solution was delivered using a 26-gauge micro syringe (Hamilton, USA). After injection, craniotomy holes were covered with styptic power (Kwik stop, ARP laboratory). The scalp was repositioned using tissue adhesive (3M Vetbond, 3M) and ketorolac was administered subcutaneously at a dose of 5 mg per kg body weight. Pups were then returned to their foster mother (female CD1 mouse) until subsequent steps in our experimental protocol. Although protocols by other groups recommend 14 days viral incubation post-injection[10], our experimental paradigm required only 4 days of incubation for robust viral labeling (Supplementary fig. 1b). For adenoviral injections involving hypoxic groups, we used a slightly modified neonatal hypoxia treatment (10.5% O_2 from P3 through P7).

At 4 and 14 days post-injection, animals in each group were anesthetized with Isoflurane and trancardially perfused with 4% paraformaldehyde solution. Brains were dissected out and post-fixed overnight in PBS. This was followed by incubation with 30% sucrose solution overnight. 40 μm thick coronal cerebellar slices were prepared using a vibratome (Thermo Scientific Microm HM 430). Cerebellar slices were first saturated with 20% goat serum for an hour and then incubated overnight with Rabbit anti-Calbindin antibody (1:2000 concentration, catalog no. CB-38a, Swant, Switzerland) on a shaker. Slices were then washed thrice with PBS, followed by secondary incubation with Alexa Fluor conjugated AffiniPure Goat Anti Rabbit IgG (Jackson Immuno Research code 111-605-144) for two hours at room temperature. Next step was phosphate buffer washing and the slices were mounted on slide with DAPI containing mounting solution (DAPI Fluoromount-GSouthern Biotech, USA). Low magnification (20X) images were obtained using a BX61 fluorescent microscope (Olympus). Virus-infected cells appeared red under green (λ=587nm) excitation light due to mCherry fluorescence. In the representative figures, anti-Calbindin labeled cells were pseudocolored as yellow (Supplementary fig. 1b). Both 4 days and 14 days post-virus-injection groups contained 3 animals each. From each mouse, eight sections (each section 40 μm thick) were randomly picked, and three 1 mm^2 areas were counted under the microscope for virus expression from each slice. Virus expression was defined as the percentage of $mCherry^+$ PCs which also co-label as anti-$Calbindin^+$ (Supplementary fig. 1c). Our quantification confirmed that only 4 days of post-injection incubation period was sufficient for ~60% virus expression in PCs of *Pcp2-cre* mice. Comparison of mean percentages of $mCherry^+$/anti-$Calbindin^+$ double-labeled cells at 4 days

and 14 days post-injection was statistically not significant (Supplementary fig. 1c, Two sample t test, t Statistic = -0.83851, DF = 17, Prob>|t| = 0.41338). Finally, to identify the extent of non-specific viral labeling of cells in the cerebellar cortex, we counted mCherry+/calbindin- cells in the molecular layer. Confirming an earlier report[11], we did notice non-specific viral expression in non-PC cells in the molecular layer in both Nx and Hx groups (Supplementary fig. 3 a,b, arrowhead). However, using our particular injection protocol which targeted the simple lobule, this non-specific labeling was exceedingly rare (2 non-PCs:133 PCs from n = 3 animals in Nx and 4 non-PCs:161 PCs from n = 3 animals; < 0.1 cells per $(100\ \mu m)^3$ in Nx and < 0.2 cells per $(100\ \mu m)^3$ in Hx;) and not significantly different between Hx and Nx groups (Supplementary fig. 3c, Unpaired t-test, two-tailed, t = 0.7686, df = 4, *P* = 0.4850; Supplementary fig. 3d, Fisher's exact test, two-tailed, *P* = 0.6938).

In a recent paper referenced above, the authors crossed the Pcp2-Cre Mpin line with the ChR2-EYFP line, and showed evidence for direct and robust photocurrents in 21% of MLIs in Pcp2-Cre-Mpin-ChR2-EYFP mice, indicating non-specificity in photoresponse to blue light stimulation[11]. As seen in Supplementary fig. 4, using *in vitro* optogenetics, we did not notice any light-evoked photoresponses in MLIs.

Since we obtained robust mCherry reporter expression in PCs of *Pcp2-cre* mice, we proceeded with optogenetic experiments combined with *in vivo* extracellular multielectrode array recordings from virus-injected mice at ages P13, P21, P30, and P45 in both Nx and Hx groups. For the pharmacological intervention group, a separate set of Nx and Hx mice at P30 and P45 were used. The stereotactic placement of electrodes is shown marked with red dots in Supplementary fig. 1d (right, image) in a coronal section of mouse cerebellum (Mouse Brain Library: C57BL/6J Coronal – Section 26)[12].

In a set of calibration experiments with *in vivo* stimulation and recording from cerebellar PCs of *Pcp2-cre* mice, we tested a range of stimulation protocols that included photo-evoked spike activity at 1 Hz, 5 Hz, 10 Hz, 15 Hz, 20 Hz, 25 Hz, 30 Hz, 40 Hz up to 100 Hz (Supplementary fig. 1e). In order to determine the optimal stimulation paradigm[13], we used stimulation duration from 1 ms to 100 ms (Supplementary fig. 1f). From this calibration, we concluded that cerebellar PCs with ChR2 displayed high fidelity (> 80%) with 25 Hz 473 nm blue LED pulses of 30 ms duration (Supplementary fig. 1g). Thus, we applied 25 pulses of blue light with a pulse width of 30 ms, and an inter-pulse interval of 10 ms [25 pulses × (30 + 10) = 1000 ms total optostimulation period] as part of our optimized paradigm for all *in vivo* optogenetics experiments.

Stimulation and simultaneous *in vivo* extracellular recording were acquired as represented in Supplementary fig. 1d, using 40 KHz OmniPlex D (version 1.11, Plexon, USA) neural data acquisition system, and preamplified using MiniDigi preamplifier (16 channels, Plexon USA). Recorded spike trains were then sorted offline using a combination of waveform template matching and primary component analysis (PCA) cluster method in Offline Sorter (version 3.0, Plexon, USA), and further analyzed in Neuroexplorer version 5.0 (Plexon, USA). PC simple spikes and complex spikes were detected independently. We performed firing frequency analysis over a two second time window (one second before and one second after optical stimulation) for all groups. For firing frequency analysis, only those PCs which had a mean post-optostimulation firing rate (optostimulation onset [t = 0] to 1000 ms) higher than the threshold defined as mean pre-optostimulation frequency (-1000 ms to optostimulation onset) + 1 standard deviation (S.D.) were selected. Regularity of PC spike firing over a time-period of 12

seconds was measured as coefficient of variance (CV). The CV was calculated as a ratio of the S.D. of interspike interval (ISI) to the mean of ISI of a given cell. Short term rhythmicity was measured as CV2 as per the following previously published[14] equation:

$$CV2 = 2\langle ISI_{i^{th}+1} - ISI_{i^{th}}\rangle / \langle ISI_{i^{th}+1} + ISI_{i^{th}}\rangle$$

For each PC, ISI values were normalized to the maximum ISI to avoid the effect of inter-PC differences in firing rates. For spike profile analysis of individual simple spikes, we plotted normalized distributions of spike duration, and slopes of depolarization and repolarization spike phase recorded from all units in each experimental group. Gaussian fits were estimated for each distribution. For complex spike detection, we used a voltage threshold applied to the primary spike in high-band-pass-filtered data[15]. Following automated sorting, each complex spike was further verified by visual inspection for spike shape, and the presence of spikelets. Firing pattern analysis was performed similar to analysis for simple spike data.

In vitro slice electrophysiology

Slice electrophysiology was performed using standard procedures[16]. Briefly, P21-P25 mCherry-ChR2-AAV injected and non-injected *Pcp2-Cre* mice were anaesthetized with isoflurane and perfused with ice-cold sucrose-based slicing buffer aerated with 95% O_2 and 5% CO_2. The buffer was composed of (in mM): 2 $MgSO_4.7H_2O$, 2 $MgCl_2.6H_2O$, 2 KCl, 1 $CaCl_2.2H_2O$, 25 NaHCO3, 1.25 NaH2PO4, 234 Sucrose and 10 glucose. Following decapitation, serial coronal slices of the cerebellum (250-300 µm thick) were obtained using a vibratome (LEICA VT1200 S). Slices were immediately transferred into a holding chamber containing ACSF composed of 125 NaCl, 2 KCl, 1 $CaCl_2.2H_2O$, 2 $MgSO_4.7H_2O$, 25 NaHCO3, 1.25 NaH_2PO4, 19.87 Sucrose and 10 glucose. The ACS was bubbled with 95% O_2 and 5% CO_2, and initially maintained at 32°C for 30 minutes for slices to recover, and subsequently at room temperature for the duration of the recording (5 h after recovery).

Following recovery, cerebellar slices were placed in a submersion type recording chamber and continuously perfused with aerated ACSF containing 125 NaCl, 2 KCl, 2 $CaCl_2.2H_2O$, 1 $MgSO_4.7H_2O$, 25 NaHCO3, 1.25 NaH_2PO4, and 25 glucose. mCherry$^+$ PCs and mCherry$^-$ MLIs were distinguished using fluorescent light and subsequently visualized under differential optics microscopy using 60× objective of a BX51W1 upright microscope (Olympus); mCherry$^-$ PCs were visualized under differential optics. Whole-cell patch-clamp recordings of PC and MLI somata were performed using pulled borosilicate glasses (5-6 MΩ) filled with internal solution composed of: 135 K-gluconate, 10 KCl, 2 MgCl2. $6H_2O$, 10 EGTA, 10 HEPES, 2 Na-ATP, 0.2 Na3 GTP, pH 7.3 (280-290mosM). An Alexa Fluor™ 488 hydrazide, sodium salt (Molecular probes) was added to the internal solution at a 1:1000 dilution to allow for posthoc detection and analysis of recorded cells. Recordings were performed using standard paradigms in both current and voltage clamp modes on Axopatch MultiClamp 700B (Molecular Devices, Sunnyvale, CA) and signals were filtered at 2 kHz and digitized (DIGIDATA 1322A, Molecular Devices) and analyzed offline using Clampfit software (Molecular Devices).

After obtaining whole-cell recording, we delivered a brief (10ms) or longer (several seconds) pulse of high-intensity LED light (470nm, Mightex, Pleasanton, CA) to stimulate PCs. The LED light was delivered via the optical path of the BX51W1 upright microscope.

SUPPLEMENTARY NOTES

PCs are less excitable to current injection in Hx mice

In our *in vitro* data, although we didn't find significant differences in current responses between virus-injected and non-injected Nx and Hx groups (Supplementary fig. 2b-d), we observed a difference in the injected current to elicited firing frequency relationship between Nx and Hx (Supplementary fig. 2e-f). We compared the best exponential fit [$Y = Y0e^{(kX)}$] of current-frequency data from Nx and Nx + ChR2 PCs and observed that data from both groups were fitted to a single exponential curve (black curve, Supplementary fig. 2e) since the Y0 and k constants in the individual fits were not significantly different between these groups. Similarly, Hx and Hx + ChR2 groups also were fitted to a single exponential curve (red curve, Supplementary fig. 2f). Thus, the current-frequency within Hx and Hx + ChR2, or Nx and Nx + ChR2 groups does not change on the basis of ChR2 expression. However, when we compared between Nx and Hx groups, we observed that the current-frequency fit constants are significantly different from each other (Extra sum-of-squares F test, $F_{(2,512)} = 10.75$, $P < 0.0001$).

PC spike profiles are altered in Hx mice and partially rescued following Tiagabine treatment

PC spike profiles are important indicators of cerebellar-cortex function and consequently locomotor behavior. As a corollary, abnormalities in PC spike profiles are potentially related to cerebellar dysfunction and locomotor deficits[17, 18]. Therefore, we performed an analysis of PC simple spike profiles from P13 and P21 mice (Supplementary fig. 6,a-d, see below). In P13 Nx mice, mean spike duration is distributed across two peaks at 0.93 ms and 1.25 ms (Supplementary fig. 6b, black). At P21, only one peak is visible in Nx mice at 0.29 ms (Supplementary fig. 6b, blue). However, in the P21 Hx group, two peaks are seen – at 0.49 ms and 0.93 ms (Supplementary fig. 6b, wine). The slower mean spike duration for the first peak, and the presence of a second peak at 0.93 ms suggests a spike profile developmentally similar to P13 Nx group. Similarly, depolarization phase slopes for Hx P21 (Supplementary fig. 6c, wine) are closer to Nx P13 (black), than to Nx P21. However, repolarization slopes of spikes are not substantially different between Hx P21 and Nx P21, when each is compared to Nx P13 (Supplementary fig. 6d). Taken together, our analysis of *in vivo* PC basal firing patterns, and spike profile analysis at P13 and P21 indicates that Hx results in a profound dysmaturation of electrophysiological properties of PCs during postnatal development.

Spike profile analysis indicated that PC simple spikes detected in Tiagabine-treated animals have a profile that is somewhat improved compared to Hx animals (Supplementary fig. 6e, Tiagabine-treated Hx animals, green line – mean profile, light green – representative profiles; compare to Hx animals, red line – mean profile, light red – representative profiles, and Nx animals, black line – mean profile, light gray – representative profiles). Mean spike duration in Tiagabine-treated Hx animals indicates the presence of two peaks at 0.35 ms and 0.56 ms – likely indicative of a partial shift towards a normal profile (Supplementary fig. 6f, compare to Nx peak at 0.26 ms and Hx peak at 0.64 ms). Similarly, repolarization phase slopes are closer to Nx animals in Tiagabine-treated Hx animals (Supplementary fig. 6h), however, depolarization slopes are shifted to the right of Hx animals (Supplementary fig. 6g).

SUPPLEMENTARY DISCUSSION

A major caveat regarding our misstep data is the contribution of brain regions other than the cerebellum, such as the brainstem, limbic system, spinal cord, basal ganglia, and cerebral cortex[19] in modulating locomotor behavior. Since our animal model is a global hypoxia model aiming to recapitulate most of the clinical hallmarks of neonatal birth injury, multiple brain regions associated with locomotor behavior could be potentially altered. Interestingly, while many studies identify the cerebellum as being particularly vulnerable during the perinatal brain injury time window, other locomotor-related brain regions such as the brainstem[20] and pre-motor regions[21] have also been suggested to undergo modest structural alterations associated with prematurity. Likely non-cerebellar candidate brain regions affected by neonatal brain injury could most likely comprise the major basal ganglia nuclei (caudate, putamen, nucleus accumbens, and pallidum) and the mesencephalic locomotor region (MLR) of the brainstem, which have long been implicated in locomotor control. In fact, a recent longitudinal study indicates that smaller volumes of basal ganglia nuclei in very preterm infants are associated with long-term neurodevelopmental motor deficits at 7 years of age[22]. This study also showed association of smaller neonatal thalamic volumes with motor deficits, suggesting that the entire cerebello-thalamo-cortical pathway[23] may be disrupted due to neonatal injury. Thus, although we cannot exclude the possibility that other brain regions might also be involved in the locomotor abnormalities observed in Hx mice, the cerebellum is an important component of the locomotor control circuit and plays a fundamental role in regulating this behavior.

While locomotion itself involved the recruitment of multiple regions of the CNS, adaptive responses during locomotion are cerebellar-dependent. In our conditioned learning paradigm (Figure 1b, c), we have set the delay between CS and US to 250ms. This timing is important to measure “true” adaptive cerebellar learning. Classical eyeblink conditioning experiments have measured the percentage of conditioned learning to peak at a delay of ~250ms[24], which is well correlated to single PC firing rates in trained adult mice[25].

In our study, we also tracked behavioral changes across two different ages. We noticed that juvenile mice are faster at adapting to an obstacle on the ladder than older mice (compare mean session 5 steptime in Figure 3a and Figure 4a). In contrast to our results, a human study using the cerebellar-dependent split belt treadmill paradigm suggested that timing of adaptation is slower in young children when compared to adolescents and adults[26]. These opposite results could be due to a host of different reasons – including inter-species differences, or differences in task design. Nevertheless, conclusions from our study regarding Nx mice more broadly agree with the literature on decreasing functional neural plasticity in specific brain regions as animals mature[27, 28].

In addition to changes in adaptive learning measured using steptime, we also noticed changes in stepping patterns in Hx mice across age (Figure 5). One possible way to explain differential changes in stepping patterns in Hx mice may have to do with risk-taking behavior, which has been correlated to cerebellar activation in fMRI studies[29, 30]. Risk-taking behavior spikes at adolescence and then trails off into adulthood[31]. P45 Nx mice (equivalent to adolescence/young adulthood) therefore use a smaller percentage of short steps since this is correlated with higher risk[32]. In comparison, P45 Hx mice display higher percentage of short steps. This result is in line with human studies measuring outcomes for young adults born prematurely, where risk-taking

behavior was markedly less pronounced[33]. Thus, reductions in risk-taking, and putatively correlated stepping patterns may be part of a larger “preterm phenotype” rather than solely due to cerebellar dysfunction[34].

In terms of pathophysiology of the cerebella of Hx animals, our observation of reduction in simple spike activity may also be related to increase in spike width in Hx animals (Supplementary fig. 6), as has been observed in PCs of mice lacking fast-acting voltage gated potassium channels[35]. Finally, in addition to changes in spike discharge and ionic equilibrium, morphological alterations may also drive behavioral changes. Since Hx disrupts the early postnatal timeline, developmental phenomena such as PC dendritic arborization may be a contributing factor. In our previous report, we observed a marked delay in dendritic arborization of PCs of Hx animals[1]. Previous work by another group has implicated PC dendrite maturation as a contributing factor to spontaneous firing[36]. An Hx-induced delay in dendritic arborization or maturation is thus consistent with our *in vivo* data showing a drastic reduction in spike frequency. This reduction in spike is not directly dependent on abnormal MLI function, since our analysis indicates that MLIs do not significantly and directly contribute to opto-evoked spike activity in PCs (Supplementary fig. 4 b).

In terms of complex spike firing, we noticed that optostimulation results in increased firing. While further experiments are needed to determine the exact mechanism behind this increase, our latency analysis of optostimulation evoked complex spike activity (Supplementary fig. 9) is in line with previous reports which employed ChR2-mediated stimulation in PCs, supporting the model of disinhibition of the inferior olive[37].

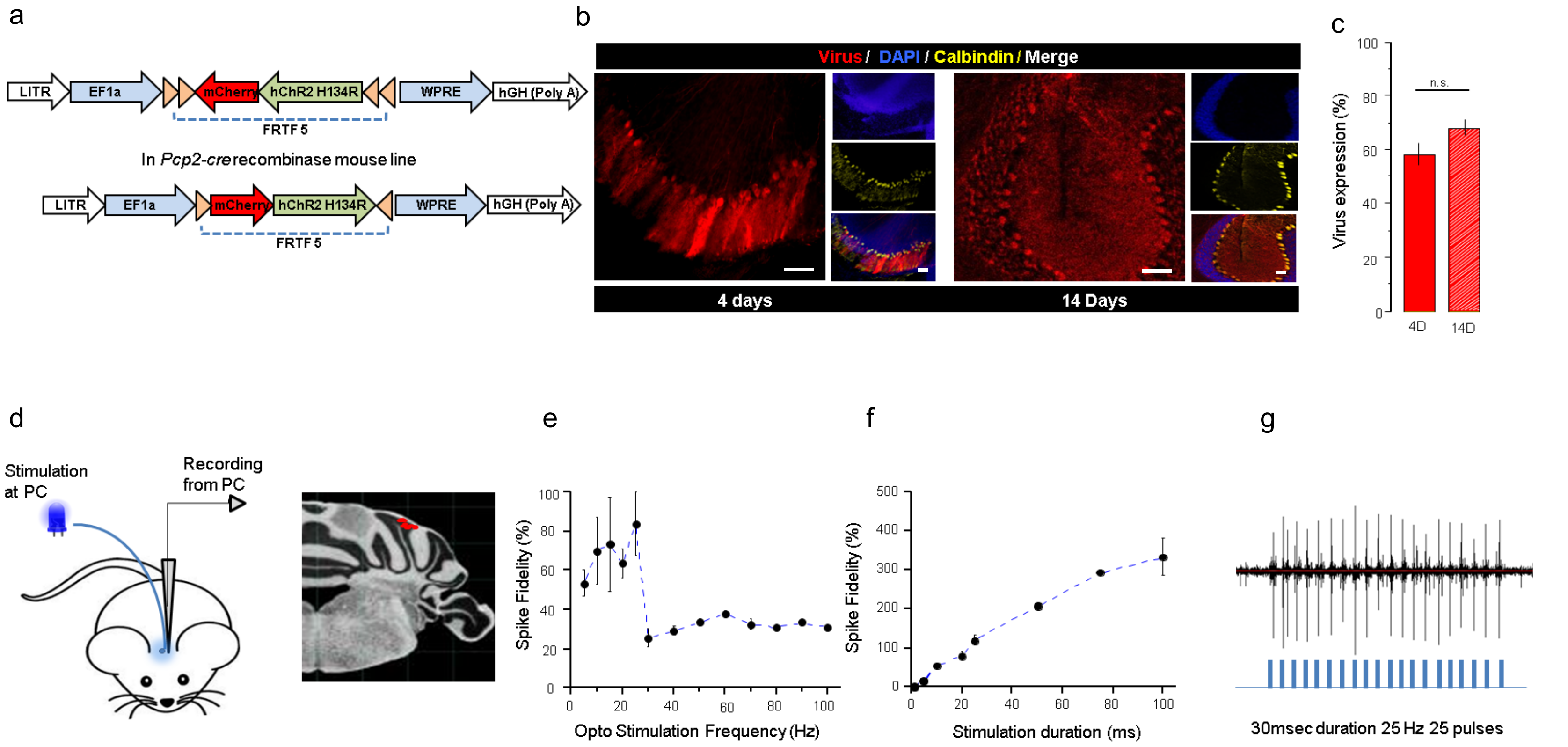

Supplementary figure 1. Validation of ChR2 adenovirus expression and optostimulation paradigm in *Pcp2 Cre* mice at juvenile stage. (a) ChR2 adeno-associated viral vector (AAV9-DIO-mCherry-ChR2) before (top) and after Cre-dependent recombination in Pcp2 Cre mouse line (bottom) causing ChR2-mCherry expression. (b) Coronal cerebellar slices showing robust ChR2-mCherry expression in PCs 4 days (left block) and 14 days (right block) following adenovirus injection (scale: 50 µm). Smaller panels show co-labeling with DAPI nuclear stain (top, blue), anti-calbindin immunohistochemistry (middle, yellow) and merged (bottom) (scale: 50 µm). (c) Bar graph comparison of percentage virus expression in PCs, 4 days (n = 15 animals) and 14 days (n = 4 animals) following adenovirus expression. Two sample t test between groups: t statistic = -0.83851, DF = 17, Prob>|t| = 0.41338, n.s.

– not significant, error bars denote SEM (d) Cartoon depicting *in vivo* multielectrode array recording coupled with optogenetic stimulation in anesthetized animals. Optostimulation and multielectrode array recordings were targeted to PCs. Inset shows coronal section of cerebellum [from mouse brain library (MBL)][112]. Red circles represent optrode position in the cerebellar cortex. (e) Spike fidelity measurement across different optostimulation frequencies and (f) stimulus (pulse width) durations (g) Representative spike recording from a single PC *in vivo* using optimized optostimulation paradigm (30 ms pulse width, inter-pulse interval = 10 ms, 25 pulses i.e. 25 Hz)

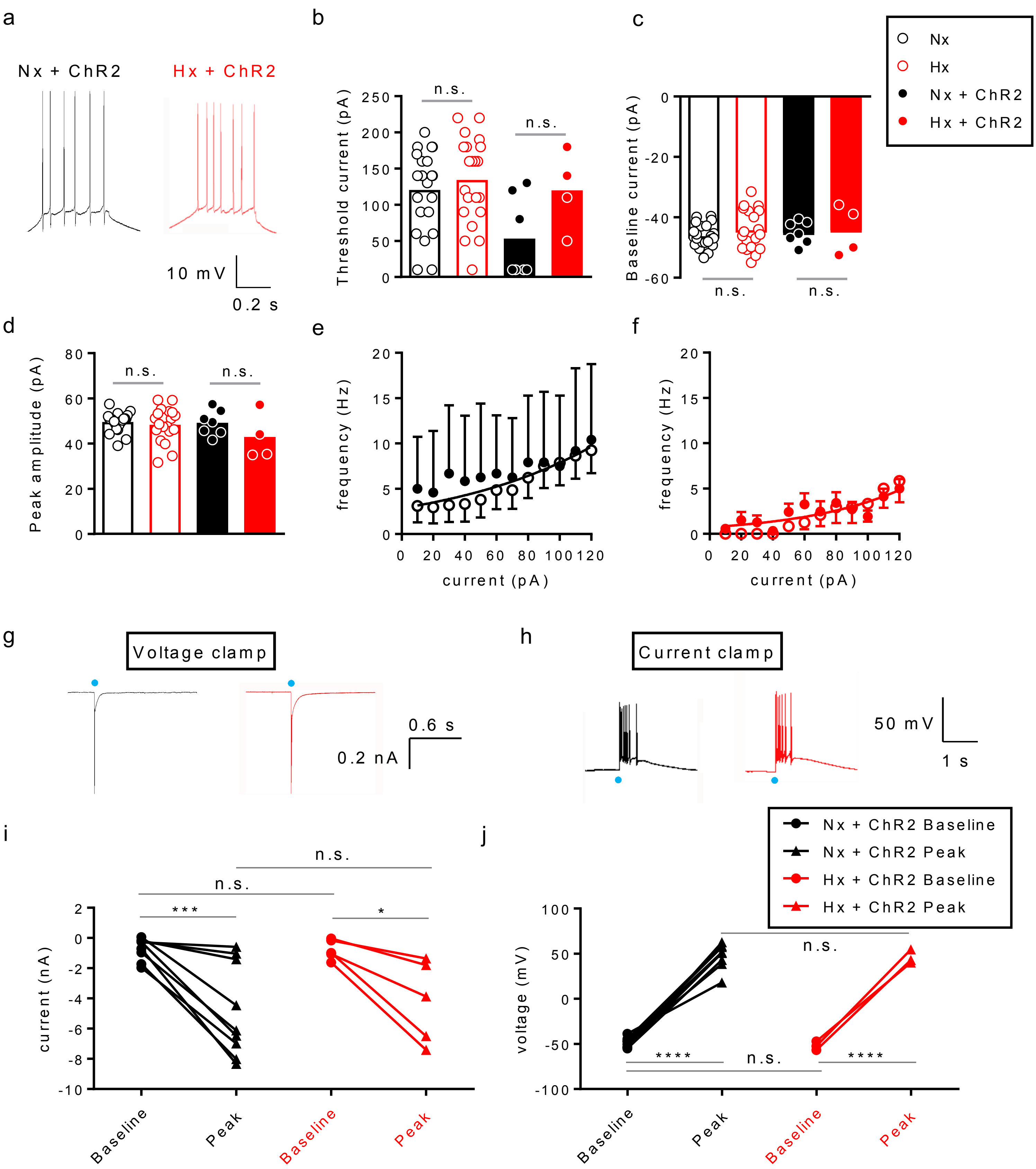
a
Nx + ChR2
Hx + ChR2
10 mV
0.2 s
b
Threshold current (pA)
n.s.
n.s.
c
Baseline current (pA)
n.s.
n.s.
Nx
Hx
Nx + ChR2
Hx + ChR2
d
Peak amplitude (pA)
n.s.
n.s.
e
frequency (Hz)
current (pA)
f
frequency (Hz)
current (pA)
g
Voltage clamp
0.6 s
0.2 nA
h
Current clamp
50 mV
1 s
i
current (nA)
***
*
n.s.
n.s.
Baseline
Peak
Baseline
Peak
j
voltage (mV)
Nx + ChR2 Baseline
Nx + ChR2 Peak
Hx + ChR2 Baseline
Hx + ChR2 Peak
n.s.
****
n.s.
****
Baseline
Peak
Baseline
Peak

Supplementary figure 2 (continued from previous page). ***In vitro*** **electrophysiological analysis of PC viability and ChR2 optostimulation.** (a) Representative ChR2-expressing PC spiking (black –Nx PC, red –Hx PC) in response to current injection (b) Scatter-bar plot comparing threshold current (c) baseline current (d) Peak amplitude of current response across all groups (black – Nx, red – Hx, open circles – non-injected animals, solid circles – ChR2-injected animals, bar represents mean) (e) Injected current to firing frequency plot in Nx PCs; current-frequency relationship for PCs in ChR2-injected (solid) and non-injected (open) slices are fitted to a shared exponential fit: $f = 2.844 \times e^{(0.01018 \times I)}$. Hypothesis testing was performed using Extra sum-of-squares F test. The hypothesis that the two groups had significantly different fitting parameters was rejected ($F_{(2,368)} = 0.5733$, $P = 0.5642$) (f) Injected current to firing frequency plot in Hx PCs; current-frequency relationship in PCs in ChR2-injected (solid) and non-injected (open) slices are fitted to a shared exponential fit: $f = 0.7245 \times e^{(0.01581 \times I)}$. The hypothesis that the two groups had significantly different fitting parameters was rejected ($F_{(2,296)} = 1.232$, $P = 0.2933$). (g) Representative photoevoked current responses to 10 ms blue light stimulation (blue solid circle) in ChR2-mCherry-expressing Nx PCs (black) and Hx PCs (red) under voltage clamp mode (h) Representative photoevoked voltage responses under current clamp mode (i) Comparison of baseline (circle) and peak (triangle) current responses under optostimulation (j) Comparison of baseline and peak voltage response under optostimulation. For b-f: Nx (n = 21-23 PCs from 3 animals), Hx (n = 19-21 PCs from 3 animals). For b-j: Nx + ChR2 (n = 7-9 PCs from 3 animals), Hx + ChR2 (n = 3-5 PCs from 3 animals). Statistical tests represented in b-d are One-way ANOVA followed by Tukey's multiple comparison test. Statistical tests represented in i-j are Two-way ANOVA followed by Sidak's multiple comparison test. Asterisks represent * $P < 0.05$, *** $P < 0.001$, **** $P < 0.0001$, 'n.s.' is not significant. Error bars in e-f represent SEM.

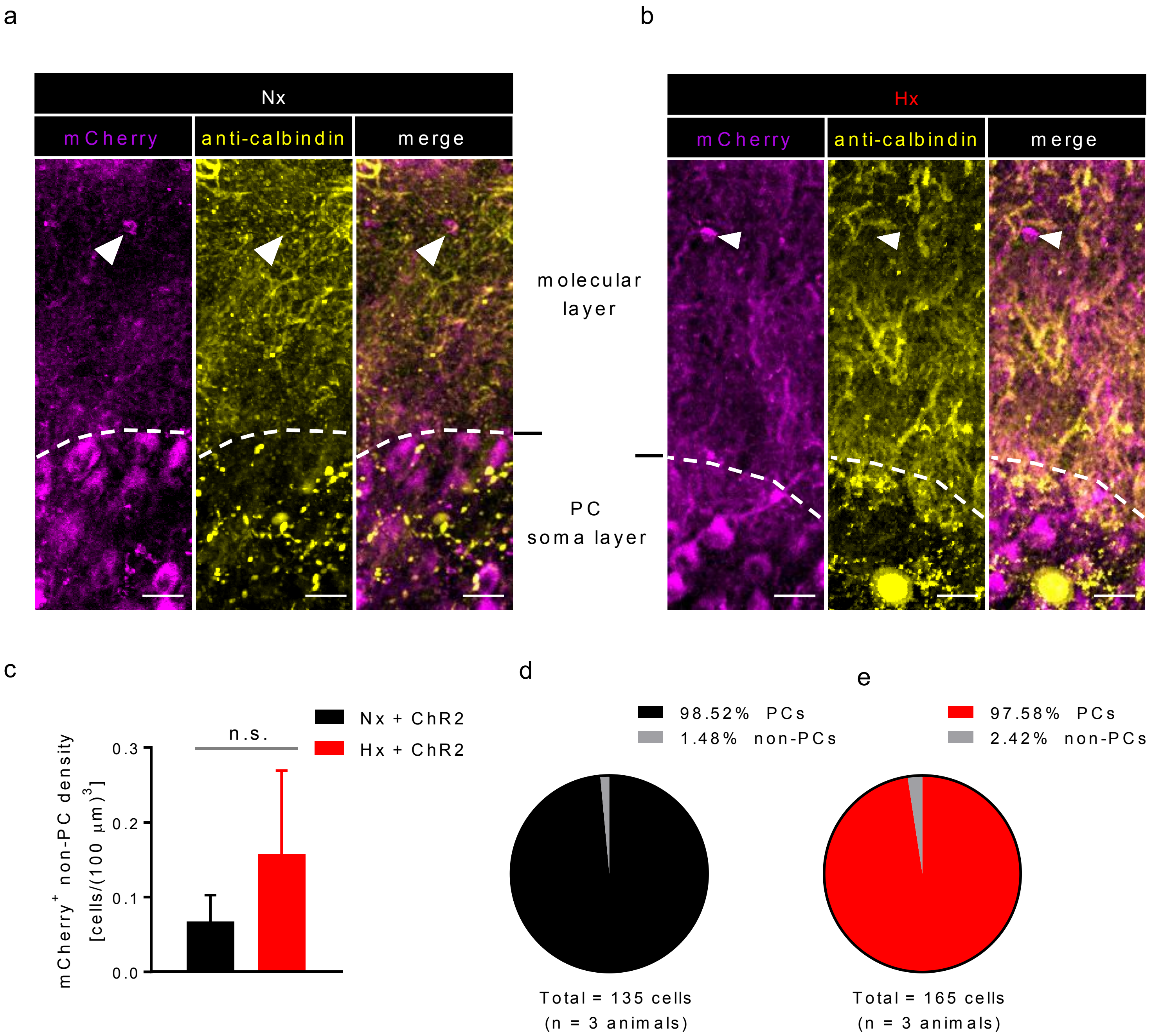

Supplementary figure 3. Immunohistological analysis of non-specific viral labeling in *Pcp2-Cre* animals injected with mCherry-ChR2 AAV. (a) Representative confocal images of 150 µm thick cerebellum sections from P21 Nx and (b) P21 Hx Pcp2-Cre animals injected with mCherry-ChR2 AAV; In panels ‘a’ and ‘b’: mCherry signal (magenta, left image), anti-calbindin antibody (yellow, middle image), and merge (right image) arrowhead points to a putative mCherry^{+} non-PC in the molecular layer (c) Cell density comparison of putative mCherry^{+} non-

PCs (n = 3 animals per group) (d) Pie chart showing percentages of PCs to non-PCs from a total sample of 135 cells (n = 3 animals) in Nx and (e) 165 cells (n = 3 animals) in Hx. Statistical test represented in panel ‘c’ is Unpaired t-test, two-tailed, $t = 0.7686$, $df = 4$, $P = 0.4850$. Statistical test performed between PC:non-PC ratio in panel ‘d’ and ‘e’ was Fisher’s exact test, two-tailed, $P = 0.6938$. n.s. is not significant. Scale in panels a-b is 25 μm.

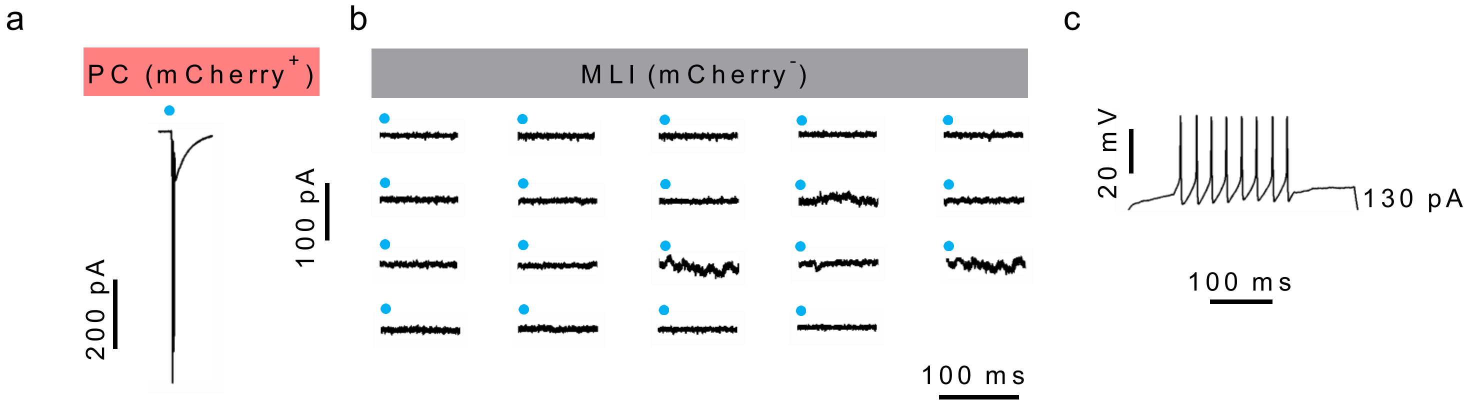

Supplementary figure 4. Optostimulation using a specific AAV-Channelrhodopsin strategy does not evoke MLI photocurrents in the Pcp2-Cre Mpin line. (a) Representative trace from slice electrophysiology showing a direct and robust photocurrent in mCherry^{+} PCs in response to blue light stimulation (blue circle). (b) Individual traces of all MLIs recorded (mCherry^{-}, 19 cells from 5 animals) in the vicinity of mCherry^{+} PCs showing the absence of photocurrent in response to blue light stimulation (blue circle). (c) Representative spikes under current clamp mode in an MLI in response to current injection (130 pA step) to ensure cell integrity and viability.

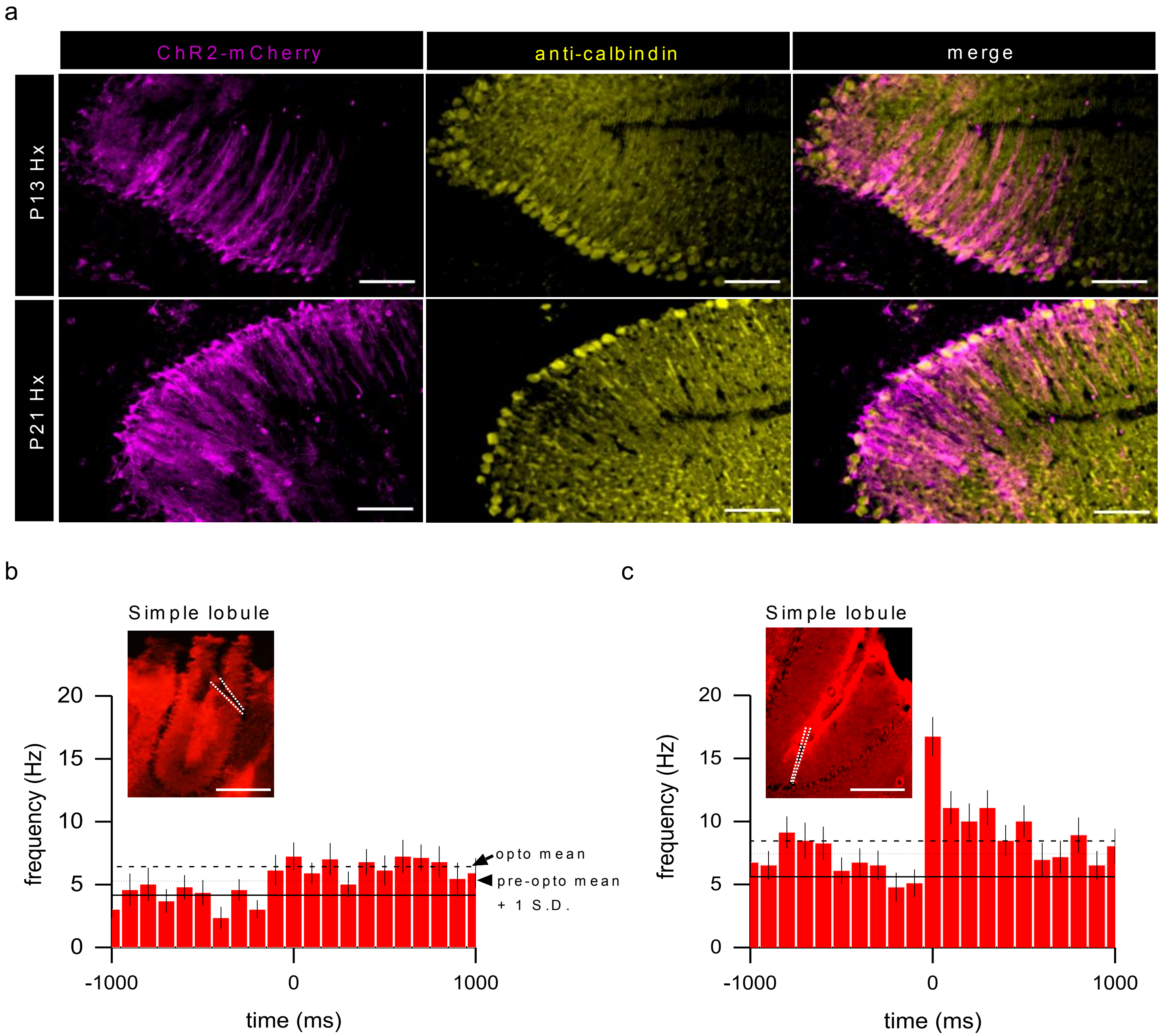

Supplementary figure 5. mCherry-ChR2 expression and Above-threshold evoked PC firing in P13 and P21 Hx animals. (a) ChR2-mCherry AAV fusion protein expression (magenta) in PCs of *Pcp2-Cre* Hx animals at P13 (left, top) and P21 (left, bottom), PC-specific anti-calbindin (anti-CB) labeling (yellow, middle, top and bottom), and merge (right, top and bottom) (c) Data from Figure 6 (c, d) replotted in smaller x and y scale to demonstrate above-threshold (pre-opto mean + 1 S.D.) evoked PC simple spike firing in P13 Hx and (d) P21 Hx

animals. Insets in panels ‘b’ and ‘c’ show multielectrode probe position (white dotted line) in the simple lobule where all virus injections and recordings were performed. Scale: ‘a’ - 100 µm, inset in ‘b’ – 200 µm.

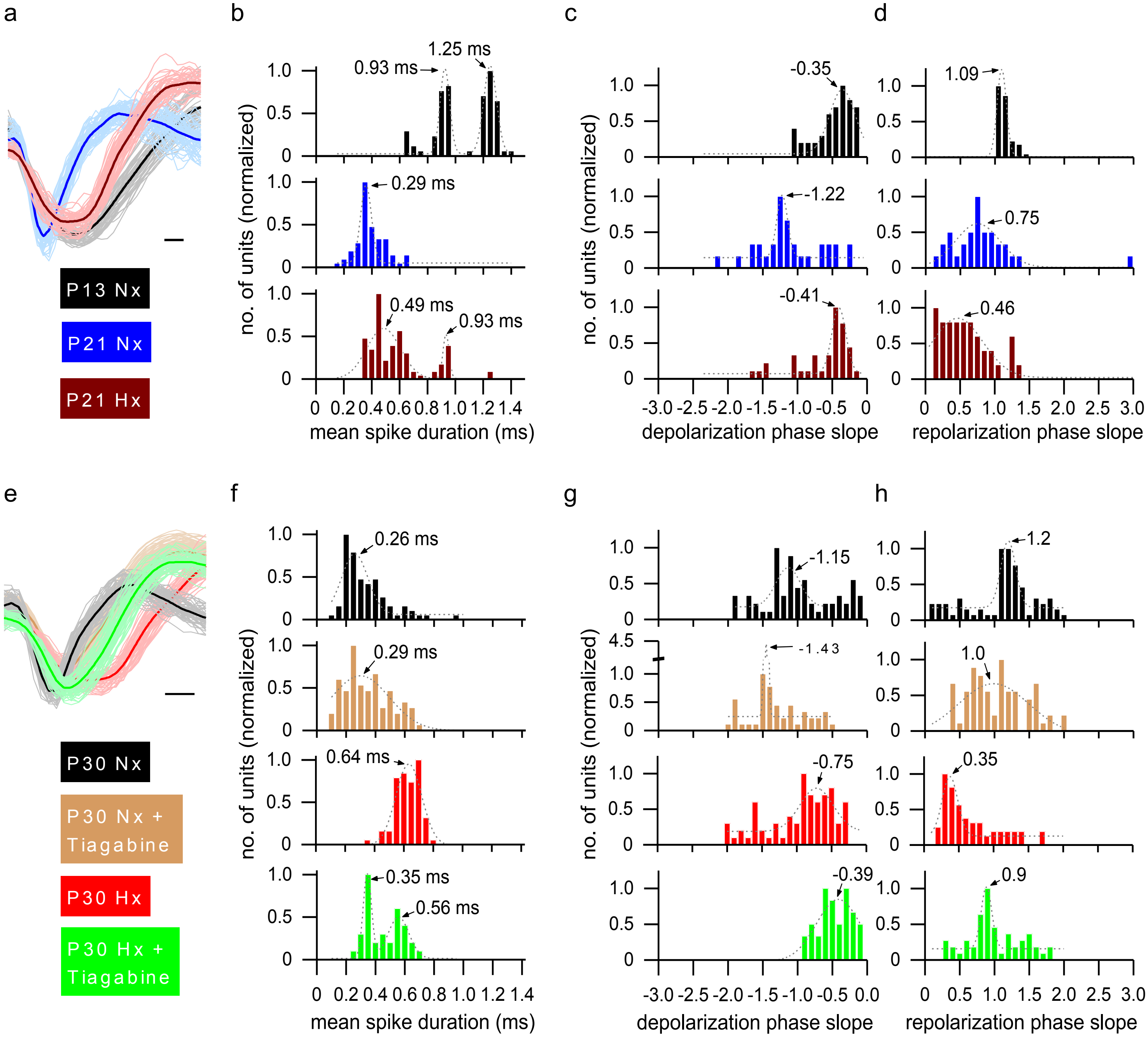

Supplementary figure 6. Spike profile analysis for PC simple spikes from P13, P21, and P30 groups. (a) Representative spikes from P13 Nx (legend: black, black trace represents mean spike profile of light gray traces from representative PC), P21 Nx (legend: blue, mean – blue trace, individual profiles – light blue) and P21 Hx (legend: wine, mean – wine trace, individual profiles – light red). (b) Normalized population distributions of mean spike durations

obtained from average simple spike profiles from multiple PCs from P13 Nx group (black bars), P21 Nx group (blue bars), and P21 Hx group (wine bars). Gray dashed line represents Gaussian fit of data with peak midpoints marked. (c) Normalized population distributions of depolarization phase slope and (d) repolarization phase slope for P13 and P21 groups. (e) Representative spikes from P30 Nx (legend: black, black trace represents mean spike profile of light gray traces from representative PC), Nx treated (legend: light brown, mean – light brown trace, individual profiles – light tan), Hx untreated (legend: red, mean – red trace, individual profiles – light red), Hx treated (legend: green, mean – green trace, individual profiles – light green). (f) Normalized population distributions of mean spike durations obtained from average simple spike profiles from multiple PCs from P30 Nx group (black bars), P30 Nx treated group (light brown bars), P30 Hx untreated group (red bars), and P30 Hx treated group (green bars). Gray dashed line represents Gaussian fit of data with peak midpoints marked. (g) Normalized population distributions of depolarization phase slope and (h) repolarization phase slope for P30 groups.

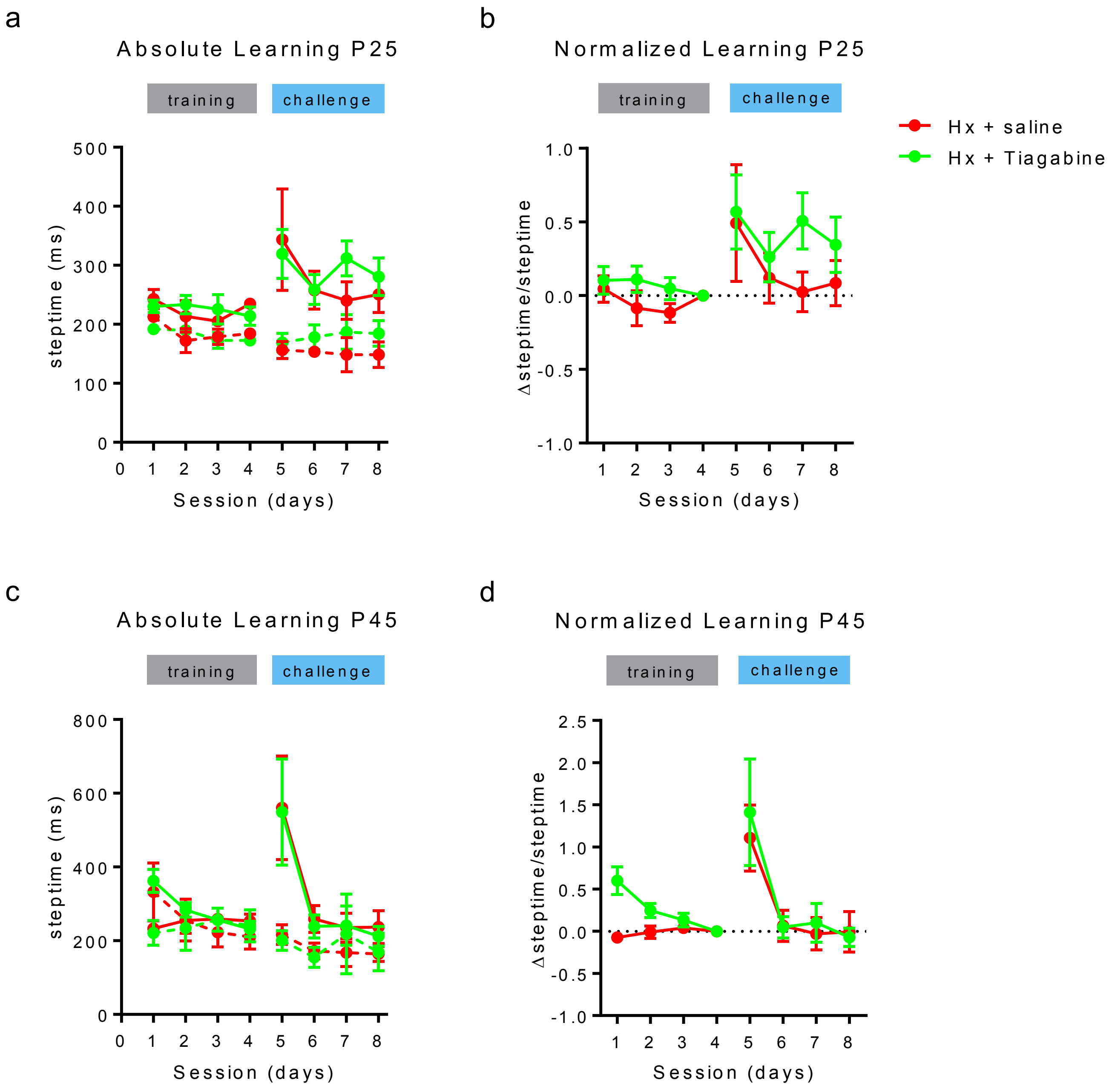

Supplementary figure 7. Pharmacological intervention using Tiagabine does not rescue adaptive cerebellar learning deficits in P25 and P45 Hx mice. (a) Absolute learning in P25 Tiagabine treated (green traces, Hx + Tagabine, n = 6) and P25 controls (red traces, Hx + saline, n = 5). Post-perturbation steptimes (solid line), pre-perturbation steptimes (dashed lines). Statistics: Two-way repeated measures (RM) ANOVA, matching: stacked along sessions,

intervention × session effect $F_{(21,140)} = 2.869$, $P = 0.0001$, session effect $F_{(7,140)} = 8.224$, $P < 0.0001$, intervention effect $F_{(3,20)} = 5.521$, $P = 0.0063$ (b) Normalized learning in P45 treated and control mice. Post-perturbation steptimes per session per mouse normalized to post-perturbation steptimes during session 4 for each mouse in treated (green) and control groups (red). (c) Absolute learning in P45 Tiagabine treated (green traces, n = 6) and P45 controls (red traces, n = 6). Post-perturbation steptimes (solid line), pre-perturbation steptimes (dashed lines). (b) Normalized learning in P45 treated and control mice. Post-perturbation steptimes per session per mouse normalized to post-perturbation steptimes during session 4 for each mouse in treated (green) and control groups (red). Statistics: Two-way repeated measures (RM) ANOVA, matching: stacked along sessions, intervention × session effect $F_{(7,70)} = 0.5995$, $P = 0.7542$, session effect $F_{(7,70)} = 7.646$, $P < 0.0001$, intervention effect $F_{(1,10)} = 1.622$, $P = 0.2316$. Tukey's multiple comparison test revealed no statistically significant differences between Tiagabine-treated and control Hx groups ($P > 0.05$) comparing post-perturbation steptimes within a single session.

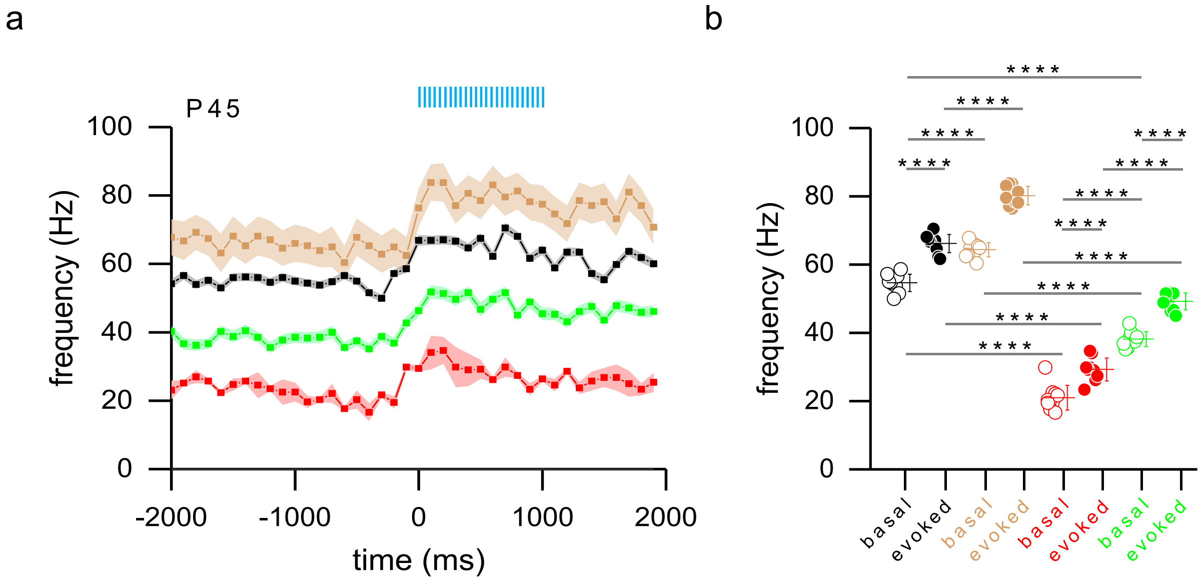

Supplementary figure 8. Reduction in basal and evoked firing in Hx animals at P45. (a) Mean firing frequency of PC SSs in P45 *Pcp2-Cre* mice from untreated Nx group (black squares and connecting line, n = 25 PCs, SEM denoted in light grey), Tiagabine-treated Nx group (light brown, n = 25 PCs SEM - light tan), untreated Hx group (red, n = 25 PCs, SEM - light red), and Tiagabine-treated Hx group (green, n = 25 PCs, SEM - light green) obtained from 8-10 animals/group. (b) Scatter plot SS firing frequency comparison across groups. For all groups, open circles represent basal firing and solid circles represent evoked (optostimulated) firing. Each circle represents mean firing frequency obtained from multiple cells per group for one sample.. Horizontal lines in each group represent the mean firing frequency across time-samples. Error bars in panels b denote SEM. Statistics: One-way ANOVA, $F_{(7,72)} = 529.5$, $P < 0.0001$, $R^2 = 0.9809$. Asterisks represent results of Tukey's multiple comparison test following One-way ANOVA **** $P < 0.0001$.

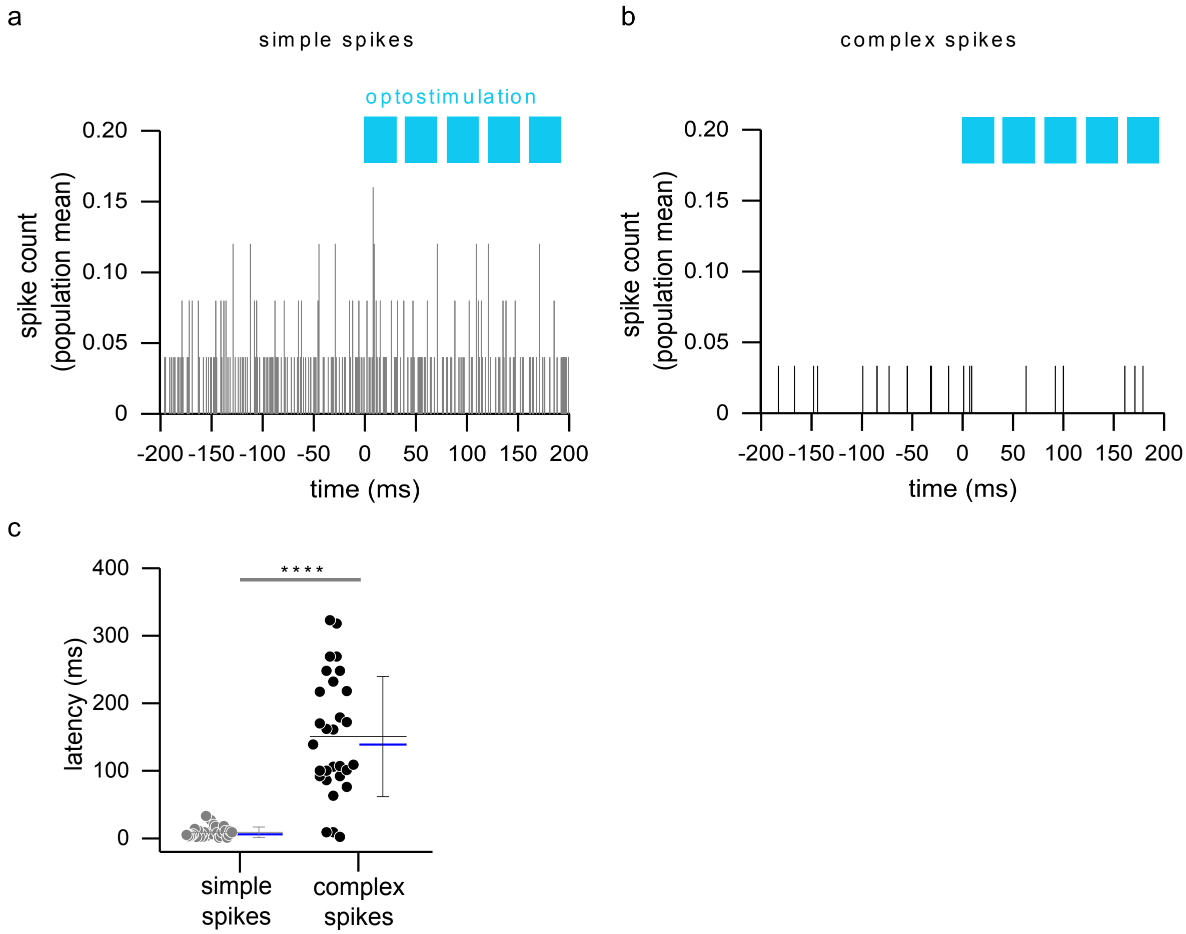

Supplementary figure 9. Evoked CSs have increased latency compared to evoked SSs. (a) Population mean spike counts for PC SSs at P30 (grey columns). 'X' axis has been plotted with a temporal resolution of 1 ms for the window -200 ms to +200 ms where t = 0 is onset of optostimulation (blue bars) (b) Population mean spike counts for PC CSs at P30 (black columns). (c) SS (grey) and CS (black) scatter plot comparison of latency to first spike after optostimulation onset. Long black horizontal line denotes mean latency; whiskers denote ± SEM (n = 29 PCs for 8-10 animals for both groups in all panels); blue line denotes median latency: SS = 6 ms, CS = 139 ms. Asterisk represents *P* < 0.0001 (exact) for Two-tailed Mann-Whitney U test. Difference between medians – Actual = 133, Hodges-Lehmann = 133.

Supplementary references

1. Zonouzi M*, et al.* GABAergic regulation of cerebellar NG2 cell development is altered in perinatal white matter injury. *Nature neuroscience* **18**, 674-682 (2015).

2. Scafidi J*, et al.* Intranasal epidermal growth factor treatment rescues neonatal brain injury. *Nature* **506**, 230-234 (2014).

3. Bi B*, et al.* Cortical glial fibrillary acidic protein-positive cells generate neurons after perinatal hypoxic injury. *The Journal of neuroscience : the official journal of the Society for Neuroscience* **31**, 9205-9221 (2011).

4. Salmaso N, Dominguez M, Kravitz J, Komitova M, Vaccarino FM, Schwartz ML. Contribution of maternal oxygenic state to the effects of chronic postnatal hypoxia on mouse body and brain development. *Neuroscience letters* **604**, 12-17 (2015).

5. Biran V, Verney C, Ferriero DM. Perinatal cerebellar injury in human and animal models. *Neurology research international* **2012**, 858929 (2012).

6. Vinueza Veloz MF*, et al.* Cerebellar control of gait and interlimb coordination. *Brain structure & function*, (2014).

7. Van Der Giessen RS*, et al.* Role of olivary electrical coupling in cerebellar motor learning. *Neuron* **58**, 599-612 (2008).

8. Saab AS*, et al.* Bergmann glial AMPA receptors are required for fine motor coordination. *Science* **337**, 749-753 (2012).

9. Rahmati N*, et al.* SLC26A11 (KBAT) in Purkinje Cells Is Critical for Inhibitory Transmission and Contributes to Locomotor Coordination. *eNeuro* **3**, (2016).

10. Kruse W, Krause M, Aarse J, Mark MD, Manahan-Vaughan D, Herlitze S. Optogenetic modulation and multi-electrode analysis of cerebellar networks in vivo. *PloS one* **9**, e105589 (2014).

11. Witter L, Rudolph S, Pressler RT, Lahlaf SI, Regehr WG. Purkinje Cell Collaterals Enable Output Signals from the Cerebellar Cortex to Feed Back to Purkinje Cells and Interneurons. *Neuron* **91**, 312-319 (2016).

12. Rosen GD*, et al.* The Mouse Brain Library, www.mbl.org. Int Mouse Genome Conference (2000).

13. Jiang L*, et al.* Cholinergic Signaling Controls Conditioned Fear Behaviors and Enhances Plasticity of Cortical-Amygdala Circuits. *Neuron* **90**, 1057-1070 (2016).

14. Holt GR, Softky WR, Koch C, Douglas RJ. Comparison of discharge variability in vitro and in vivo in cat visual cortex neurons. *J Neurophysiol* **75**, 1806-1814 (1996).

15. Warnaar P*, et al.* Duration of Purkinje cell complex spikes increases with their firing frequency. *Frontiers in cellular neuroscience* **9**, 122 (2015).

16. Abbah J, Braga MF, Juliano SL. Targeted disruption of layer 4 during development increases GABAA receptor neurotransmission in the neocortex. *Journal of neurophysiology* **111**, 323-335 (2014).

17. Raman IM, Sprunger LK, Meisler MH, Bean BP. Altered subthreshold sodium currents and disrupted firing patterns in Purkinje neurons of Scn8a mutant mice. *Neuron* **19**, 881-891 (1997).

18. Walter JT, Alvina K, Womack MD, Chevez C, Khodakhah K. Decreases in the precision of Purkinje cell pacemaking cause cerebellar dysfunction and ataxia. *Nature neuroscience* **9**, 389-397 (2006).

19. Takakusaki K. Neurophysiology of gait: from the spinal cord to the frontal lobe. *Movement disorders : official journal of the Movement Disorder Society* **28**, 1483-1491 (2013).

20. Kim H*, et al.* Hindbrain regional growth in preterm newborns and its impairment in relation to brain injury. *Human brain mapping* **37**, 678-688 (2016).

21. Karolis VR*, et al.* Reinforcement of the Brain's Rich-Club Architecture Following Early Neurodevelopmental Disruption Caused by Very Preterm Birth. *Cereb Cortex* **26**, 1322-1335 (2016).

22. Loh WY*, et al.* Neonatal basal ganglia and thalamic volumes: very preterm birth and 7-year neurodevelopmental outcomes. *Pediatric research* **82**, 970-978 (2017).

23. Horne MK, Butler EG. The role of the cerebello-thalamo-cortical pathway in skilled movement. *Progress in neurobiology* **46**, 199-213 (1995).

24. Ohyama T, Nores WL, Murphy M, Mauk MD. What the cerebellum computes. *Trends in neurosciences* **26**, 222-227 (2003).

25. ten Brinke MM*, et al.* Evolving Models of Pavlovian Conditioning: Cerebellar Cortical Dynamics in Awake Behaving Mice. *Cell reports* **13**, 1977-1988 (2015).

26. Patrick SK, Musselman KE, Tajino J, Ou HC, Bastian AJ, Yang JF. Prior experience but not size of error improves motor learning on the split-belt treadmill in young children. *PloS one* **9**, e93349 (2014).

27. Ito M. 'Nurturing the brain' as an emerging research field involving child neurology. *Brain & development* **26**, 429-433 (2004).

28. Anderson V, Spencer-Smith M, Wood A. Do children really recover better? Neurobehavioural plasticity after early brain insult. *Brain : a journal of neurology* **134**, 2197-2221 (2011).

29. Ernst M*, et al.* Decision-making in a risk-taking task: a PET study. *Neuropsychopharmacology : official publication of the American College of Neuropsychopharmacology* **26**, 682-691 (2002).

30. Cardoso Cde O, Branco LD, Cotrena C, Kristensen CH, Schneider Bakos DD, Fonseca RP. The impact of frontal and cerebellar lesions on decision making: evidence from the Iowa Gambling Task. *Frontiers in neuroscience* **8**, 61 (2014).

31. Laviola G, Macri S, Morley-Fletcher S, Adriani W. Risk-taking behavior in adolescent mice: psychobiological determinants and early epigenetic influence. *Neuroscience and biobehavioral reviews* **27**, 19-31 (2003).

32. Espy DD, Yang F, Bhatt T, Pai YC. Independent influence of gait speed and step length on stability and fall risk. *Gait & posture* **32**, 378-382 (2010).

33. Hack M, Flannery DJ, Schluchter M, Cartar L, Borawski E, Klein N. Outcomes in young adulthood for very-low-birth-weight infants. *The New England journal of medicine* **346**, 149-157 (2002).

34. Johnson S, Marlow N. Preterm birth and childhood psychiatric disorders. *Pediatric research* **69**, 11R-18R (2011).

35. Hurlock EC, McMahon A, Joho RH. Purkinje-cell-restricted restoration of Kv3.3 function restores complex spikes and rescues motor coordination in Kcnc3 mutants. *The Journal of neuroscience : the official journal of the Society for Neuroscience* **28**, 4640-4648 (2008).

36. Womack M, Khodakhah K. Active contribution of dendrites to the tonic and trimodal patterns of activity in cerebellar Purkinje neurons. *The Journal of neuroscience : the official journal of the Society for Neuroscience* **22**, 10603-10612 (2002).

37. Chaumont J*, et al.* Clusters of cerebellar Purkinje cells control their afferent climbing fiber discharge. *Proceedings of the National Academy of Sciences of the United States of America* **110**, 16223-16228 (2013).
